# HIPPIE: a new platform for ambient-pressure X-ray photoelectron spectroscopy at the MAX IV Laboratory

**DOI:** 10.1107/S160057752100103X

**Published:** 2021-02-12

**Authors:** Suyun Zhu, Mattia Scardamaglia, Jan Kundsen, Rami Sankari, Hamed Tarawneh, Robert Temperton, Louisa Pickworth, Filippo Cavalca, Chunlei Wang, Héloïse Tissot, Jonas Weissenrieder, Benjamin Hagman, Johan Gustafson, Sarp Kaya, Fredrik Lindgren, Ida Källquist, Julia Maibach, Maria Hahlin, Virginia Boix, Tamires Gallo, Foqia Rehman, Giulio D’Acunto, Joachim Schnadt, Andrey Shavorskiy

**Affiliations:** a MAX IV Laboratory, Lund University, Box 118, 221 00 Lund, Sweden; b Division of Synchrotron Radiation Research, Department of Physics, Lund University, Box 118, 221 00 Lund, Sweden; c Department of Physics, Tampere University of Technology, PO Box 692, FIN-33101 Tampere, Finland; d Material Physics, School of Engineering Sciences, KTH Royal Institute of Technology, 100 44 Stockholm, Sweden; e Department of Chemistry, Koc University, Istanbul 34450, Turkey; f Department of Physics and Astronomy, Division of Molecular and Condensed Matter Physics, Uppsala University, 751 20 Uppsala, Sweden; g Institute for Applied Materials, Karlsruhe Institute of Technology, Hermann-von-Helmholtz-Platz 1, 76344 Eggenstein-Leopoldshafen, Germany; h Department of Chemistry – Ångström Laboratory, Uppsala University, Box 538, 751 21 Uppsala, Sweden

**Keywords:** APXPS, *operando*, *in situ*, synchrotron, catalysis, IR, beamline

## Abstract

HIPPIE is a soft X-ray beamline on the 3 GeV electron storage ring of the MAX IV Laboratory, equipped with a novel ambient-pressure X-ray photoelectron spectroscopy (APXPS) instrument. The endstation is dedicated to performing *in situ* and *operando* X-ray photoelectron spectroscopy experiments in the presence of a controlled gaseous atmosphere at pressures up to 30 mbar [1 mbar = 100 Pa] as well as under ultra-high-vacuum conditions. The photon energy range is 250 to 2200 eV in planar polarization and with photon fluxes >10^12^ photons s^−1^ (500 mA ring current) at a resolving power of greater than 10000 and up to a maximum of 32000. The endstation currently provides two sample environments: a catalysis cell and an electrochemical/liquid cell. The former allows APXPS measurements of solid samples in the presence of a gaseous atmosphere (with a mixture of up to eight gases and a vapour of a liquid) and simultaneous analysis of the inlet/outlet gas composition by online mass spectrometry. The latter is a more versatile setup primarily designed for APXPS at the solid–liquid (dip-and-pull setup) or liquid–gas (liquid microjet) interfaces under full electrochemical control, and it can also be used as an open port for *ad* *hoc*-designed non-standard APXPS experiments with different sample environments. The catalysis cell can be further equipped with an IR reflection–absorption spectrometer, allowing for simultaneous APXPS and IR spectroscopy of the samples. The endstation is set up to easily accommodate further sample environments.

## Abstract 2

The setup and specifications of the new soft X-ray beamline HIPPIE, dedicated to ambient-pressure X-ray photoelectron spectroscopy at the MAX IV Laboratory, are summarized. Several scientific examples are also discussed to prove the capabilities of the endstation.

## 1. Introduction   

Ambient-pressure X-ray photoelectron spectroscopy (APXPS) is a powerful technique for studying the chemical composition of the interfaces between solids, liquids and gases. The method has increased in popularity in recent years, and many experimental systems have been installed both in home laboratories, making use of X-ray anodes, and at synchrotron radiation facilities (Starr *et al.*, 2013 ▸; Arble *et al.*, 2018 ▸; Schnadt *et al.*, 2020 ▸). Currently almost all synchrotron light sources either have or are planning to have an instrument capable of measuring X-ray photoelectron spectroscopy (XPS) at mbar pressures [1 mbar = 100 Pa]. The majority of such instruments employ a back-filling scheme for creating ambient-pressure (AP) conditions in the analysis chamber. Recently, new developments have also demonstrated the high potential of an approach based on an exchangeable AP cell design for experiments in non-standard or highly demanding environments (Grass *et al.*, 2010 ▸; Velasco-Vélez *et al.*, 2016 ▸; Held *et al.*, 2020 ▸).

At the former MAX-lab (which has evolved into the current MAX IV Laboratory) a new cell-in-cell approach was pioneered (Schnadt *et al.*, 2012 ▸) as a natural development of the very early schemes of APXPS instrumentation (Siegbahn & Siegbahn, 1973 ▸; Joyner & Roberts, 1979 ▸; Joyner *et al.*, 1979 ▸; Boronin *et al.*, 1988 ▸). In this concept, which was developed simultaneously for X-ray anode-based instruments, the advantages of back-filling and exchangeable cell design are combined to allow quick switching between AP and ultra-high-vacuum (UHV) conditions without compromising sample cleanness. This is made possible by placing an AP cell inside the UHV vessel separated from the main analysis volume by a valve. The cell can then be moved by means of a motor into the analysis volume and ‘docked’ onto the front of the analyser in just a few minutes. In this setup an AP created *inside* the docked cell does not drastically compromise the vacuum in the analysis chamber, and a quick restoration of UHV conditions is possible after removal of the cell.

The first cell-in-cell approach was realized at the SPECIES beamline at MAX-lab, which is now installed on the 1.5 GeV storage ring of the MAX IV Laboratory (Schnadt *et al.*, 2012 ▸; Urpelainen *et al.*, 2017 ▸; Kokkonen *et al.*, 2021 ▸). SPECIES is a beamline that is optimized for the ultraviolet and soft X-ray range with photon energies from around 30 eV up to *ca* 1500 eV, which is ideal for the study of the valence band and the *K*-edge spectra of low-*Z* elements. In contrast, HIPPIE was designed to give access to the upper end of the soft X-ray range with a high and rather constant photon flux up to 2000 eV and additional access to the P *K* edges at a more limited flux. At the same time, a design criterion for HIPPIE was that C *K*-edge X-ray absorption spectroscopy (XAS) should be possible, which required a minimum energy of around 250 eV.

HIPPIE serves a large number of different user communities, with most users coming from the surface science and catalysis domains. In order to satisfy the very different needs of the user communities, which also include *e.g.* energy generation and storage, electrochemistry, corrosion, oxide and semiconductor thin film growth, liquids, geo- and biochemistry and photochemistry, a highly flexible design was chosen for the instrument, which allows quick exchange between various sample environments. The communities depending on the flow type rather than the batch type of gaseous sample environments benefit from a complex, fully automatic gas dosing system with online gas analysis hardware.

Below we describe the HIPPIE beamline in more detail. We address the design goals, characteristics and performance, and give a few examples of scientific problems solved using the capabilities of the beamline.

## 2. Design goals and technical details   

The following design goals were formulated for the HIPPIE beamline:

(i) To allow APXPS and soft X-ray XAS in the range between 250 and 2200 eV at a high flux (10^13^ photons s^−1^).

(ii) To allow APXPS at pressures up to 30 mbar with recording times of swept spectra on the minute timescale.

(iii) To allow fast acquisition of ambient-pressure X-ray photoelectron (APXP) spectra with frame rates in fixed mode of up to 120 Hz.

(iv) To allow fully automatic gas dosing of multiple gases as well as an online analysis of gas mixtures simultaneous with XPS (also at high sampling frequency).

(v) To allow quick and easy switching between UHV and AP conditions without compromising sample cleanness.

(vi) To allow a variety of different sample environments by means of different AP cells.

The energy range demand was met by installing HIPPIE on the 3 GeV storage ring of MAX IV. Its energy range is 250 to 2200 eV, and the photon flux is above 10^12^ s^−1^ (at 500 mA ring current) at a resolving power greater than 10000 between 250 and 2000 eV. Hence, the *K*-edge spectra of the important low-*Z* elements C, O, N and F are accessible, but also the *K* edges of Na, Mg and Al. Excellent conditions exist for measurement of the *L* edges of the 3*d* transition metal elements and the important semiconductor elements Ge and As. The possibility of tuning the kinetic energy across a wide range implies that depth profiling for multiple photoemission levels from all elements of the periodic table is possible. Higher kinetic energy values imply an increase in the electron inelastic mean free path (IMFP) and the accessibility of the solid–liquid interfaces and solid–gas interfaces at higher pressures.

HIPPIE has two branches that use the same source and monochromator; however, at the time of writing only one branch is in operation. The other branch is assembled up to the monochromator exit slit, and recently we received funding to finish the remaining part by 2024. The branch in operation is dedicated to APXPS on interfaces between solids, liquids and gases; the pressure range is from 1 × 10^−9^ up to 30 mbar.

### 2.1. Beamline   

#### 2.1.1. Source   

The source of the HIPPIE beamline is an APPLE-II-type elliptically polarizing undulator (EPU). Full polarization control is achieved from a design with four arrays of permanent magnets, two of which can be shifted longitudinally to create horizontal and vertical magnetic fields (Sasaki *et al.*, 1993 ▸). A period length of 53 mm was chosen to satisfy the requirement of full polarization control at a minimum energy of 250 eV, which corresponds to a minimum undulator gap of 11 mm. This dimension was driven by the size of the vacuum pipe in the storage ring. The undulator has 70 full periods and a total length of 3.9398 m. The design is based on glued magnet pairs, a concept developed at the MAX IV Laboratory for magnet holders with wedges for both transverse planes (Wallén *et al.*, 2014 ▸). The HIPPIE EPU was built and characterized in-house at the MAX IV magnetic measurement laboratory. The overall dimension of a single magnet block is 30 mm × 30 mm × 13.25 mm. The magnetic material is NdFeB with a magnetic remanence of *B*_r_ = 1.28 T for the vertically magnetized block and *B*_r_ = 1.25 T for the longitudinally magnetized block. At the moment the HIPPIE EPU is only used in planar mode due to the highest user demand. Other polarization modes (inclined and elliptical) are awaiting commissioning, in particular energy calibration.

#### 2.1.2. Optical design   

**Figure 1:**
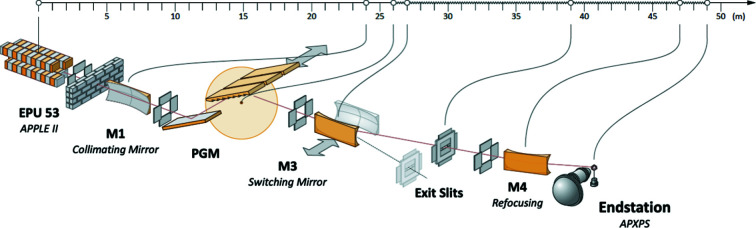
Optical layout of the HIPPIE beamline.

The layout of the optical components of the HIPPIE beamline is shown in Fig. 1 ▸, and the general details of the beamline are summarized in Table 1 ▸. The beamline design is based on a plane-grating monochromator illuminated by collimated light (cPGM), a design developed at BESSY (Follath *et al.*, 1998 ▸). The first optical component is a toroidal mirror (M1) located 24 m after the light source. It is designed to collimate the beam vertically and focus the beam horizontally onto the exit slit, 15 m further downstream. The monochromator is manufactured by Toyama and contains a plane mirror (M2) and a plane grating (PG1) with blazed profile and 1200 lines mm^−1^ density. The dispersed radiation from the monochromator is focused vertically onto the exit slit by the cylindrical mirror (M3). The refocusing for the APXPS branch is accomplished by a single toroidal mirror (M4). The focusing is astigmatic by design: the actual vertical focus is 25 mm further downstream than that of the horizontal focus, which is co-located with the sample position. The astigmatism reduces the sensitivity of the vertical beam size at the sample plane with respect to the exit slit height adjustment, which controls the energy bandwidth of the incoming radiation – this kind of focusing solution was realized and successfully tested at the SPECIES beamline (Urpelainen *et al.*, 2017 ▸). Although easy manipulation of the photon beam location at the sample is often connected to two mirror systems in a Kirkpatrick–Baez configuration (Kirkpatrick & Baez, 1948 ▸), the same can be achieved with a single mirror: here the beam spot can be moved horizontally and vertically by adjusting the pitch and roll angles of the refocusing mirror, respectively. It should be noted that, due to large deviation in the meridional (long) radius of the present M1, the horizontal focus does not meet the sample plane and the photon beam at the sample is defocused also in the horizontal direction. The first and second mirrors (M1 and M2) are internally water cooled, whereas the PG is side cooled by water-cooled copper blocks. The focusing mirror M3 is cooled through a copper brace. The refocusing mirror, M4, receives so little heat that no cooling is necessary.

A novel mirror chamber design is used for all mirrors outside the monochromator tank, *i.e.* mirrors M1, M3 and M4. This design aims to keep the weight of all components very low in order to push up the fundamental vibration frequencies, which improve stability of the beam considerably. In the design the mirror is rigidly connected to the surrounding vacuum vessel. The movement of the mirror is accomplished by moving the entire chamber by means of five motors (Agåker *et al.*, 2020 ▸). The vacuum vessel is mounted onto a granite slab which effectively brings the stability of the MAX IV experimental hall floor right below the mirror chamber. The mirror chambers do not have direct vacuum pumping; the pumping is instead accomplished via a separate pumping unit mounted on the beamline adjacent to the mirror chamber. In this way, the weight of the actual mirror chamber is reduced. The pumping unit contains an ion pump and – for M3 and M4 – a single-axis manipulator with a selection of diagnostic tools: an AXUV100G photodiode, YAG crystal and a gold mesh for drain current measurements. The X-ray beam can be shaped by four independently moving blades, each at the entrances to the monochromator tank, M3 and M4. In addition, the beam can also be shaped in the beamline’s front-end by water-cooled masks. These masks are used primarily for selection of the central part of the undulator cone and for decreasing the heat load on the beamline components.

#### 2.1.3. Beamline performance   

**Figure 2:**
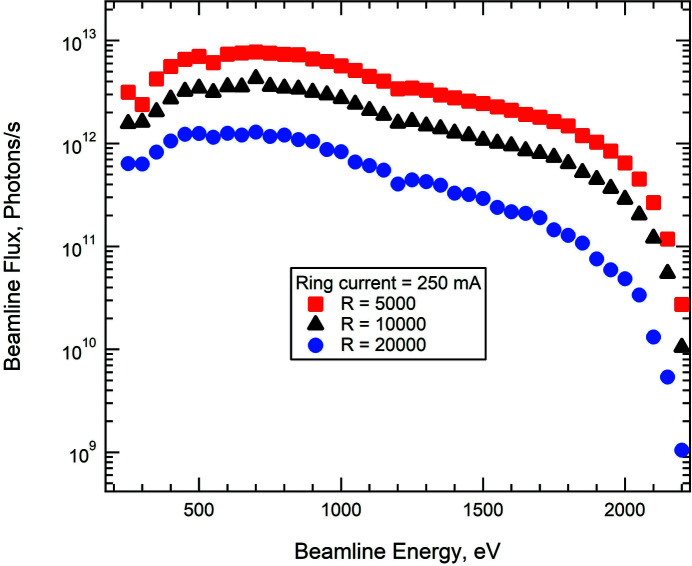
Beamline flux in the energy range from 250 to 2200 eV, measured with a ring current of 250 mA and three different resolving powers.

The photon flux at the beamline was measured using a photodiode and corrected for the photodiode’s quantum efficiency (ITW, AXUV 100G, Opto Diode, https://optodiode.com/photodiodes-axuv-detectors.html). The photodiode was mounted on a manipulator upstream of the M4 mirror. The flux was measured at 250 mA ring current, which is half of the designed value of the 3 GeV ring of MAX IV, with the undulator in horizontal linear polarization mode. Fig. 2 ▸ shows beamline flux from 250 to 2200 eV with three different resolving powers; the flux is higher than 1 × 10^12^ photons s^−1^ up to a photon energy of 1600 eV (2000 eV) with resolving power 10000 and 250 mA (500 mA) ring current.

**Figure 3:**
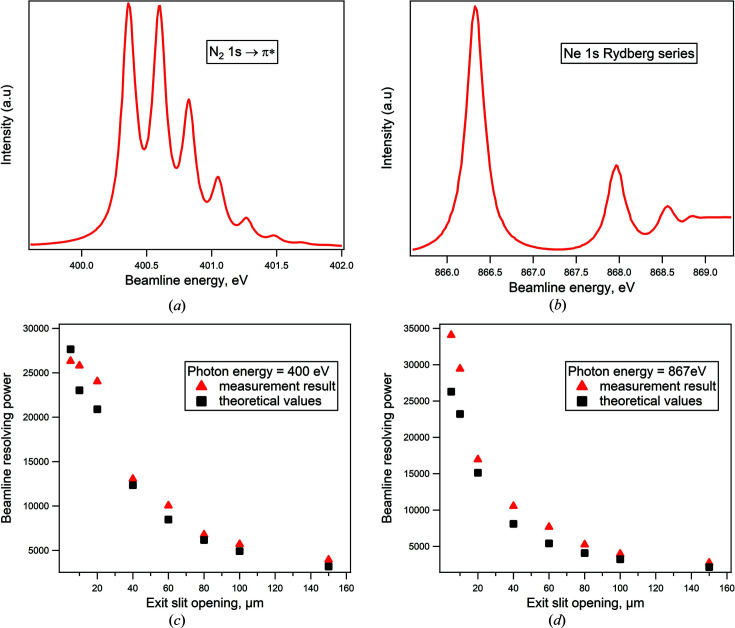
(*a*) N_2_ and (*b*) Ne *K*-edge ion yield spectra. (*c*) Resolving power of the beamline at 400 eV. (*d*) Resolving power of the beamline at 867 eV.

The resolving power was estimated from the ion yield spectra of N_2_ and Ne gases. The measurements were conducted in a gas cell downstream of the exit slit, using a gas pressure of around 1 × 10^−2^ mbar. The monochromator was operated using *c*_ff_ = cos(β)/cos(α) = 2.25, where α and β are the entrance and exit angles of the beam onto the grating, respectively. It should be noted that this *c*_ff_ value is standard for user operation mode at the beamline, and the resolving measurement presented below will thus reflect actual resolutions for users, while higher energy resolution is expected for other *c*_ff_ values. Fig. 3 ▸(*a*) shows the N *K*-edge ion yield spectrum recorded with a 5 µm exit slit opening. The beamline contribution to the line width is *ca* 16 meV, which gives a resolving power of 25800. We arrived at this result by fitting the spectrum using a Voigt profile with a Lorentzian width of 120.0 meV, a typical value found in the literature for the N 1*s* lifetime width (Feifel *et al.*, 2004 ▸; Kato *et al.*, 2007*a* ▸). The photon energy resolution is then deduced from the Gaussian broadening, and it is determined as an average Gaussian width of the four most intense vibrational peaks. Fig. 3 ▸(*b*) shows an ion yield spectrum of the Ne *K*-edge Rydberg series recorded with a 5 µm exit silt opening. We find a resolving power of 32000 from the Gaussian width of the first peak of the series of *ca* 27 meV. In the curve fit we applied a Voigt profile with a Lorentzian width of 250 meV (Kato *et al.*, 2007*b* ▸).

Figs. 3 ▸(*c*) and 3 ▸(*d*) compare the measured resolving power with the theoretical resolving power calculated through ray-tracing of the beamline. The measured resolving powers were derived from N_2_ and Ne ion yield spectra, respectively, and the ray-tracing was calculated with *RAY-UI* developed at Helmholtz Zentrum Berlin (Baumgärtel *et al.*, 2016 ▸). The ray-tracing incorporated the measured optical metrology data for the installed optical components and the same beamline settings as used in the resolving power measurement. The correspondence between the measured and theoretical values is overall good, in particular since the extraction of a Gaussian component much narrower than the Lorentzian component from a Voigt profile is difficult and seldom gives unambiguous results. It is clear, though, that the measured resolving powers indicate a beamline performance comparable with or slightly beyond the values expected from the ray-tracing.

**Figure 4:**
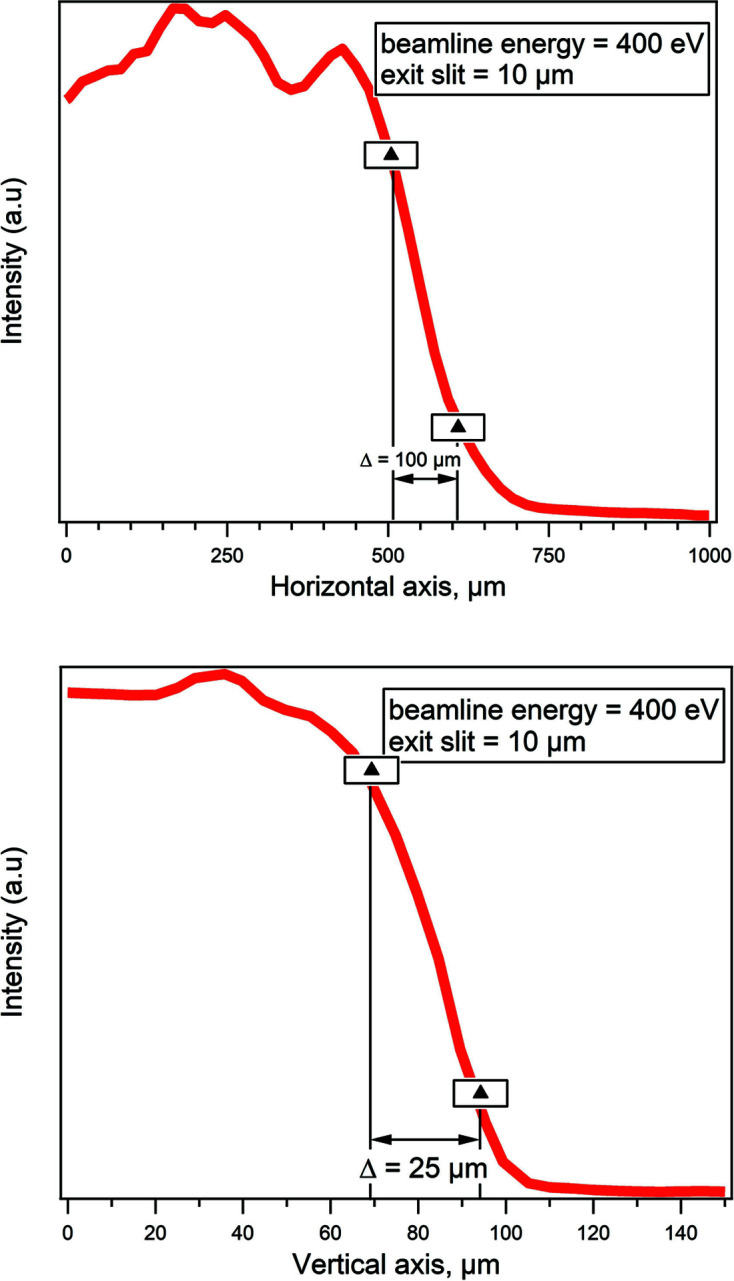
Beamline spot size at the sample surface. The beamline energy was 400 eV and the exit slit set to 10 µm in vertical size, the *Y* axis in the figure is drain current from a sharp platinum foil edge, spot size was estimated from an evaluation of the displacement needed for the drain current intensity to change from 20% to 80%, horizontal and vertical beam sizes in the figure are 100 µm and 25 µm, respectively.

The spot size at the sample plane was measured using a sharp edge of platinum foil. This edge was raster-scanned at the sample plane, and the drain current was used to measure the spot profile. The spot size at the sample was estimated from an evaluation of the displacement needed for the drain current intensity to change from 20% to 80%. Fig. 4 ▸ shows an example measured at 400 eV photon energy and a 10 µm exit slit opening. The results for the horizontal and vertical beam sizes are *ca* 100 µm and 25 µm, respectively. Considering the 35° incident angle between the photon beam and the sample surface, the beam size at the sample position in the horizontal direction is *ca* 60 µm. Both values match very well the results of the ray-tracing simulations performed including the measured figure errors of the optics.

With spherical or cylindrical optical components, small deviations in the mirror parameters from the design values can be compensated for by adjusting the incidence angle a small amount and fine-tuning the entrance and exit arms of the optical components. With this in mind, the foreseen freedom in the placement of the optical components drove the criteria set for optics procurement. However, as mentioned above, the meridional radius of the first mirror was larger, by 15%, with respect to the specification (1216580 mm versus 1057821.9 mm), and it was obvious that this cannot be compensated for. At first glance the applicability of this mirror looks very bad: the horizontal beam size at the sample plane becomes twice as large as designed. There are, however, positive aspects in that, too. Namely, the fact that the horizontal focus is no longer at the sample plane indicates that the horizontal beam size is largely dictated by the divergence of the photon beam. As the electron beam in the MAX IV 3 GeV ring has extremely low emittance, and the undulators are fairly long, the photon beam emitted by the source has very low divergence. The increase in horizontal beam size at the sample plane, although large taken relatively, is not large in absolute terms: the beam size is still measured in tens of micrometres.

It is interesting to see that even here, with a diffraction-limited storage ring, the beam properties along the beamline are further limited by diffraction. The beamline is currently operated with a reduced front-end aperture (1.7 mm × 1.9 mm) that diffracts the beam and disturbs collimation of M1 vertically. The smaller aperture results in the grating being only partly illuminated, and thus affecting the achievable resolution by the diffraction limit, which in the case of gratings is simply the number of illuminated grooves. Finally, if a very small exit slit opening is used, the vertical beam profile starts to show a typical diffraction pattern at the sample plane, which is intentionally out of focus.

### 2.2. APXPS endstation   

#### 2.2.1. General design and vacuum system   

The APXPS endstation was designed and produced by PREVAC sp. z.o.o. (https://www.prevac.eu/). The endstation is designed to allow for different sample environment solutions using both the cell-in-cell and exchangeable cell concepts. When switching between the two different concepts, the beamline entrance and electron energy analyser – a ScientaOmicron HiPP-3 analyser (Cai *et al.*, 2019 ▸) – remain in place, while the sample environments are exchanged. In addition, the endstation has a stationary UHV preparation chamber (base pressure 1 × 10^−10^ mbar), load lock chamber (1 × 10^−9^ mbar) with a sample carousel for six samples and with halogen lamps for quick bakeout, and radial distribution chamber (UFO, 1 × 10^−10^ mbar) for transfer of samples between the different parts of the system and likewise equipped with a sample storage carousel for six samples. The average time for sample transfer between any two places is less than a minute. The endstation makes use of a gas system for up to eight gases. This gas system is described in more detail below.

The ScientaOmicron HiPP-3 electron energy analyser has a differential pumping stage and electrostatic lens system that allows for AP operation at up to 30 mbar at 0.3 mm nozzle diameter. The analyser is placed horizontally, in the plane of the storage ring and at 55° with respect to the direction of the X-ray beam. The microchannel plate detector can be equipped with two different cameras with either 17 Hz or 120 Hz frame rate, which allows *ca* 60 ms and 8 ms time resolution in fixed mode, respectively. A special property of the HiPP-3 electron analyser is that spatially resolved experiments along a line are possible by inserting an additional aperture in the nozzle. In our case the spatial resolution of the analyser was measured to be 8 µm in the spatial detector direction.

An Al *K*α X-ray anode source together with monochromator is installed in the analysis chamber (Scienta­Omicron MX650), which allows measurement with online synchrotron radiation light or offline anode X-ray source, and the endstation is also designed to allow measurement of XPS in both UHV and AP conditions. For AP measurements inside the docked cell a nozzle (in standard operation 0.3 mm-diameter aperture for synchrotron light and 0.8 mm for Al *K*α X-ray anode source operation) is placed on the analyser entrance. This nozzle is not a fixed part of the analyser, but is rather part of the cell, which implies that measurements outside the cell in UHV conditions are performed with a larger analyser opening. Another special feature of the HiPP-3 analyser is the swift acceleration mode (Edwards *et al.*, 2015 ▸), which substantially improves its performance under ambient conditions, especially at low kinetic energies where electron scattering is most prominent. This mode is also the standard user mode, as it in general gives higher electron transmission than all other analyser modes.

The preparation chamber is a typical surface science preparation vacuum chamber equipped with low-energy electron diffraction (LEED, OCI BDL800IR-3G), quadrupole mass spectrometer (QMS, MKS Microvision 2, up to 200 a.m.u. [a.m.u. = atomic mass units]), quartz crystal microbalance (QCM, Prevac QO 40A1), ion source (Prevac IS 40C1), a range of ports for user equipment such as evaporators and a gas/vapour dosing system for four gases plus a noble gas for sample sputtering. The chamber manipulator is equipped with two slots for sample heating: an e-beam heater (up to 1200°C) and a low degassing resistive heater (up to 600°C). In the resistive heater slot, the sample can also be cooled to −100°C using the vapour of liquid nitro­gen. Pumping of the preparation chamber is achieved using a turbomolecular (Pfeiffer HiPace 700) and an ion getter pump (Gamma Vacuum TiTan 400L).

**Figure 5:**
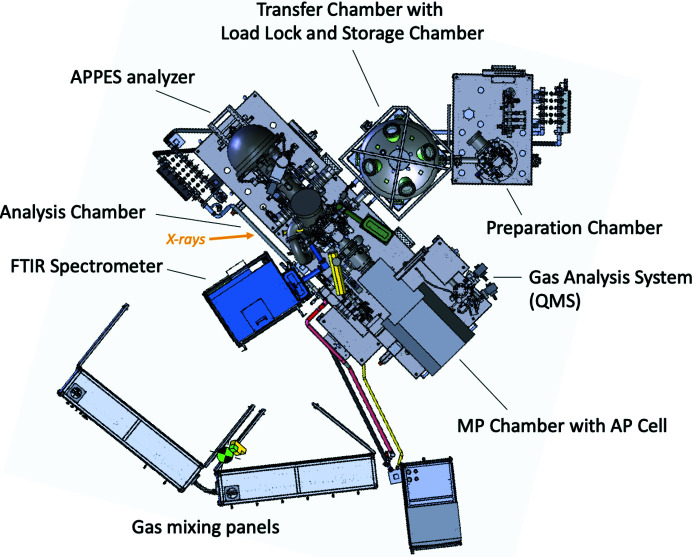
APXPS endstation at HIPPIE beamline with the catalysis AP setup.

The endstation standard setup shown in Fig. 5 ▸ is in the cell-in-cell configuration. This configuration features, in addition to the above-mentioned vacuum vessels, an analysis chamber (1 × 10^−10^ mbar), AP cell chamber (MP, 1 × 10^−9^ mbar) and a quadrupole mass spectrometer (QMS, 1 × 10^−10^ mbar) chamber. All chambers are separated from each other by gate valves. On the MP chamber a manipulator is mounted that carries the AP cell. The AP cell can be exchanged; presently, there exists one setup, the catalysis AP cell setup, which is described in more detail in Section 2.2.2.

The analysis chamber is a vacuum vessel of similar size to the preparation chamber. It features the same pumping scheme and a UHV manipulator with similar heating and cooling performance. It is connected to the endstation gas system (see below), and a Bruker Vertex 70v FTIR spectrometer can be attached to the analysis chamber for *in situ* XPS and IRRAS [IR reflection–absorption spectroscopy] measurement (see below). The MP chamber holds the AP cell. It can be valved off from the analysis chamber when doing UHV XPS measurements.

Samples are mounted on a flag-style sample holder with an on-board thermocouple connection with a design that makes it possible to image the samples both on Omicron and SPECS scanning tunnelling microscopes. Two choices of materials are available: Ta for experiments at high temperature in the absence of oxygen/water and stainless steel (304L, maximum 900°C) for all other experiments. Sample holders may have a hole in the middle for direct sample heating with the laser (see below), thus ensuring that the sample body is the hottest place in an AP experiment.

**Figure 6:**
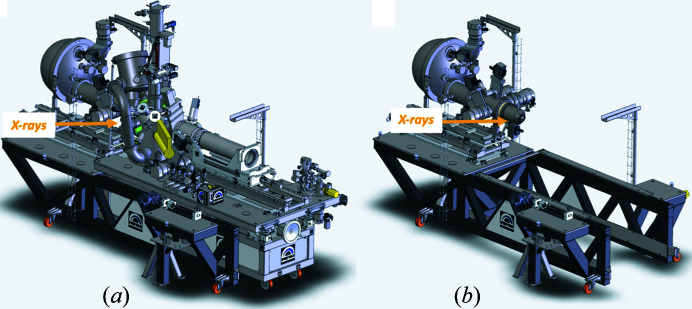
Exchanging AP cells at HIPPIE. (*a*) By the replacement of the existing AP cell. (*b*) By replacement of entire analysis and MP chambers.

The analysis and MP chambers and the AP cell manipulator are mounted on moveable rails. Using these rails, this setup can be removed (Fig. 6 ▸) and substituted by a vacuum-chamber-based AP cell setup. At present, one such setup exists: the electrochemical/liquid cell (EC), described in Section 2.2.3.

#### 2.2.2. Catalysis AP cell   

*General description*. A cell-in-cell approach already previously implemented at MAX IV is adopted for the catalysis AP cell (Schnadt *et al.*, 2012 ▸; Knudsen *et al.*, 2016 ▸). As described above, the cell is retractable. It is isolated in the MP chamber during UHV operation of the endstation and moved into the analysis chamber and docked to the electron energy analyser in AP operation. In docking mode, the analysis chamber maintains a pressure below 10^−7^ mbar when the AP cell itself is filled up to around 5 mbar. The X-ray beam from the undulator source or X-ray anode enters the AP cell through an X-ray window (200 nm silicon nitride on silicon frame). The cell also has viewports for a camera view and a visible light source.

The sample normal is aligned to the symmetry axis of the analyser electrostatic lens system. The sample can be moved laterally, while sample rotation is not possible inside the cell. The sample is heated from the back by a fibre-coupled IR laser emitting 800 nm at a maximum power of 2.8 W. Temperatures up to 600°C can be obtained in a gaseous atmosphere at a few mbars of pressure. Cooling to −10°C is possible using ethanol as chilling agent. The laser heating stage can be replaced by a ceramic heater.

*Gas delivery and gas analysis system*. The gas inlet system of the catalysis AP cell contains eight gas lines equipped with individual mass flow controllers (MFCs) (Brooks GF125 with flows up to 30 s.c.c.m. [s.c.c.m. is standard cubic centimetres per minute]). The setup allows permanent installation of standard gases such as H_2_, O_2_, CO and CO_2_, as well as user-defined gases without frequent venting and time-consuming bakeout of the gas lines. The pre-defined gas mixture from the MFCs is dosed into the AP cell, either directly or via an adjustable leak valve. This dual type of inlet systems gives our users the possibility to dose gas mixtures at any total pressure between 1.0 × 10^−8^ and 30 mbar.

The catalysis AP cell is equipped with two different pumping lines with a 4 mm inner diameter tube and three slots with opening 7 mm × 100 mm and connected to a 35 mm tube, and equipped with individually butterfly valves (VAT series 615). In a typical experiment the 35 mm pumping line is normally fully closed (by an additional in-vacuum valve at the cell) and the 4 mm pumping line partly open. In order to reach 1 mbar under these conditions a total flow of *ca* 3 s.c.c.m. is needed. Also, with these settings and a cell volume of 1 l the gas exchange time becomes 6 s. However, the adjustable pumping speed of the cell allows the user to change the gas exchange time for a given pressure. Faster gas exchange times are here achieved by increasing the flow and pumping speed.

The pressure inside the cell is measured by an in-vacuum Pirani gauge (Pfeiffer-vacuum TTR 91) for *in situ* pressure measurements between 1 × 10^−4^ and 30 mbar backed up by a combination of a capacitance manometer (pressure range from 1.33 × 10^−2^ to 133 mbar, Pfeiffer-vacuum CCR 362) and a full-range gauge (pressure range from 1 × 10^−9^ to 1 × 10^3^ mbar, Pfeiffer-vacuum PKR360) placed on the AP cell’s pumping line.

The AP cell’s inlet and outlet lines as well as the first differential pumping stage of the analyser are connected to a quadrupole mass spectrometer (Hiden HAL/3F PIC) via a combination of automatic angle and leak valves, providing a way for the quick analysis of the gas composition before or after its contact with the sample. The design avoids artificial pressure build-ups and dead volumes in the gas pipes. The users can change from probing the inlet to probing the outlet gas composition within 5 s.

**Figure 7:**
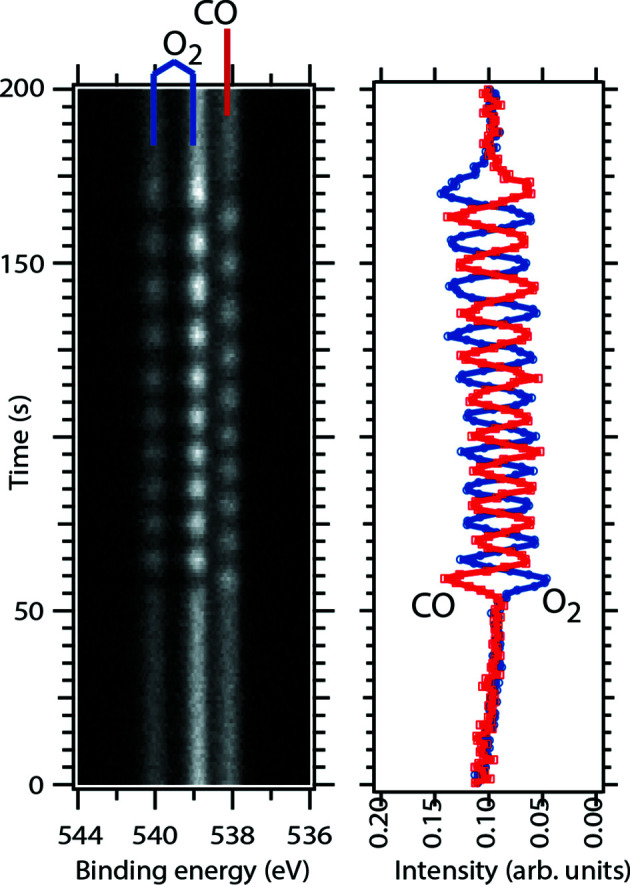
Left: O 1*s* image plot measured with ∼7 Hz and a photon energy of 650 eV and the sample slightly retracted such that no secondary electrons from the sample surface reach the electron analyser. Right: integrated CO and O_2_ signal obtained from the O 1*s* image plot displayed as a function of time.

The fast switching between gas mixtures inside the cell was demonstrated in an experiment, in which the inlet composition oscillated in a fast and reproducible fashion as illustrated in Fig. 7 ▸. The left panel shows an image plot of O 1*s* gas-phase spectrum recorded during a train of pulses of pure CO (5.5 s duration) alternating with the pulses of pure O_2_ (5.5 s duration). In total, ten such pulses were injected at 30 s.c.c.m. total flow and 1 mbar total pressure. Before and after the injection of the pulses a 1:1 CO:O_2_ composition was established at a flow rate of 15 s.c.c.m. at each mass flow controller. Gas injection started at time 0. The right panel in Fig. 7 ▸ shows the integrated signal of the CO (red) and O_2_ (blue) components. Using the 1:1 CO:O_2_ composition set by the MFCs, the integrated signals were normalized to the same intensity at time 0.

Fig. 7 ▸ illustrates that it takes *ca* 50 s at a MFC gas-flow setting of 30 s.c.c.m. before the gas pulses arrive at the sample position. Smaller total flows will result in longer waiting times. Fig. 7 ▸ also demonstrates that it is possible to maintain the shape of the gas pulse train, which is made up from pulses with different composition, for at least the 50 s it takes to reach the sample position. This observation clearly demonstrates laminar flow within the inlet line of the HIPPIE gas system.

*Polarization-modulated IR reflection–absorption spectroscopy setup*. A Bruker Vertex 70v FTIR spectrometer with all-in-vacuum beam path and polarization modulation (PM) module can be attached to the catalysis AP cell setup (blue and green components in Fig. 5 ▸), allowing simultaneous IRRAS and XPS measurements under *in situ* conditions. The IR beam first passes a polarization modulator [42 kHz PEM (AR-coated) and F350 polarizer]. Via a gold-covered plane mirror and a gold-covered out-off-axis parabolic (OAP) mirror the light is transferred into the catalysis AP cell via ZnSe viewports separating the beam path pipes, analysis chamber and catalysis AP cell, respectively. The beam illuminates the sample at 7° grazing incidence. After reflection by the sample the light goes through the same series of IR light-transmitting viewports. A single OAP mirror is used to reflect the light into the detection module with a MCT detector (medium band, spectral range 12000–600 cm^−1^). The combined wavenumber range of the system is 8000 to 720 cm^−1^ with a standard resolution of 0.4 cm^−1^. The simultaneous use of PM-IRRAS and APXPS is illustrated in Section 3.3.

#### 2.2.3. Electrochemical/liquid cell   

A dedicated cell built to use another switching approach is available for XPS measurements on liquids as well as liquid–gas and solid–liquid interfaces. The versatility of this chamber allows the design of experiments with non-standard sample environments, and it is therefore not limited only to liquid–gas and solid–liquid interfaces. This chamber is connected only to the Scienta­Omicron electron energy analyser, but not the radial distribution chamber and other parts of the endstation. The main application of the chamber is *operando* measurements on electrochemical (EC) systems and hence it is denoted as the ‘EC cell’.

The EC cell is a vacuum vessel with a background pressure of 1 × 10^−5^ mbar. It has a large loadlock-style port (door) for quick access to the chamber’s interior for sample loading. The chamber can be backfilled with gases and vapours at pressures up to ∼50 mbar (when using a 0.3 mm front cone opening), and the gaseous atmosphere can be heated to ∼90°C by halogen lamps. A four-axis manipulator (*XYZ* and rotation) can be inserted from the top, and a three-axis manipulator (*XYZ*) with cooling and heating functionality (via liquid coolant media) can be inserted from the bottom. Two top manipulators are available: one for liquid jet and one for electrochemistry experiments. A custom-designed glove­box can be attached to the EC cell and is equipped with a moisture detector (Mbraun, MB-MO_SE1) and an oxygen detector (Mbraun, MB-OX-SE1). The glovebox provides inert conditions for sample introduction and thus allows sensitive sample measurement at the endstation.

**Figure 8:**
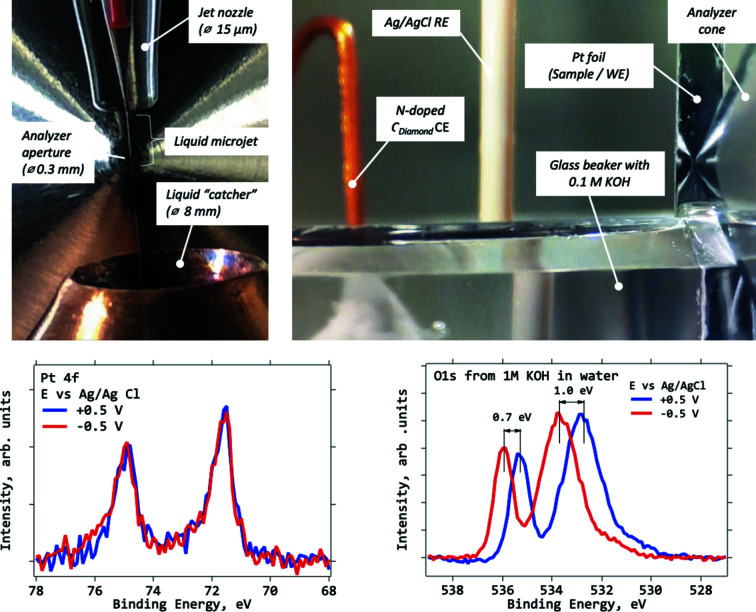
Electrochemical (EC) cell at HIPPIE beamline. Liquid microjet (top left) emerging from the end of a 20 µm glass nozzle into an opening of a ‘catcher’. In the foreground an analyser cone with 300 µm opening can be seen. Dip-and-pull setup (top right) showing sample (working electrode), Ag/AgCl reference and N-doped diamond counter-electrodes from right to left. The sample is in XPS position with the mirror reflection of the analyser cone being visible on the sample’s surface. Pt 4*f* (bottom left) and O 1*s* (bottom right) XP spectra measured in the setup shown in the top-right panel at 24 mbar of H_2_O and room temperature. Blue (red) curves correspond to +0.5 V (−0.5 V) applied potential. Thickness of KOH electrolyte layer estimated by the attenuation of Pt signal (vacuum spectrum is not shown) is *ca* 20 nm. Each spectrum took *ca* 3 min to record. *h*ν = 1800 eV, photon energy = 100 eV, exit slit 100 µm. Potentials are with respect to Ag/AgCl reference electrode.

The liquid jet manipulator provides the possibility for APXPS measurements of surfaces of liquids as well as the liquid–gas interfaces. The setup (Microliquids) contains a glass nozzle with a 15 or 20 µm opening, connected to a liquid supply pump (0.01–9.9 ml min^−1^ liquid flow) to provide a pressurized liquid flow through the nozzle (Fig. 8 ▸, top left). As the liquid is ejected from the nozzle it forms a jet which remains undisrupted for a length dependent on the liquid pressure and the opening of the nozzle. Typically, the jet is intact up to a few mm, after which it breaks down into droplets. A truncated conical copper trap (catcher) with an opening of 8 mm and a volume of 300 ml is mounted on the bottom manipulator. It collects the ejected liquid. The temperature of the collected liquid can be controlled and defines the vapour pressure in the chamber.

The electrochemical top manipulator is equipped with three electrical feedthroughs, where EC electrodes can be mounted, while the bottom manipulator is equipped with two electrical feedthroughs and is designed to hold a container for liquid electrolytes. The system is designed to carry out ‘dip-and-pull’ (also known as ‘meniscus’ method) experiments, in which up to three electrodes can simultaneously be plunged into a liquid electrolyte and retracted to allow for the XPS investigation of the liquid–solid interface during electrochemical control of the sample (Fig. 8 ▸, top right).

For a test of the probing depth and the continuity of the potential profile across the thin liquid film, a Pt foil sample and 0.1 *M* KOH solution were used. Fig. 8 ▸ shows Pt 4*f* (bottom left) and O 1*s* (bottom right) XP spectra recorded after the Pt sample was first immersed and then partially pulled out from the KOH solution. The thickness of the KOH/water film at the measurement point was estimated by the attenuation of the Pt 4*f* signal using Lambert–Beer law for XPS: 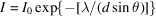 where λ is electron IMFP in liquid water (Emfietzoglou & Nikjoo, 2007 ▸), *d* is layer thickness and θ is takeoff angle (Fadley, 2010 ▸). It was found to be 20 nm. At this liquid film thickness, it took approximately 3 min to record a single Pt 4*f* spectrum using electrons of kinetic energy 1726 eV (1800 eV photon energy). Previously, the usage of ‘tender’ X-rays (3–6 keV) was considered necessary for probing the solid–liquid interfaces with XPS (Axnanda *et al.*, 2015 ▸). To our knowledge, this is the first time that the dip-and-pull method is demonstrated using soft X-rays. The O 1*s* XP spectrum demonstrates two features with the low-binding-energy component corresponding to liquid water and the high-binding-energy one to gas-phase H_2_O. Different potentials were applied to the sample with the blue (red) curves, representing spectra recorded at +0.5 V (−0.5 V). Due to the grounding of the sample the Pt spectra overlap. The O 1*s* spectra, on the other hand, undergo a shift to lower binding energy when the potential is changed from a negative to a positive value: the liquid component shifts by 1.0 eV and the gas-phase one by 0.7 eV. The equality of the shift of the liquid component and the difference in applied potentials imply that the potential is carried through the thin film without any losses. The drop occurs at the liquid–solid interface, which is normal behaviour for such systems (Shavorskiy *et al.*, 2017 ▸).

## 3. Scientific examples   

### 3.1. Following dynamic phase changes under a graphene cover   

**Figure 9:**
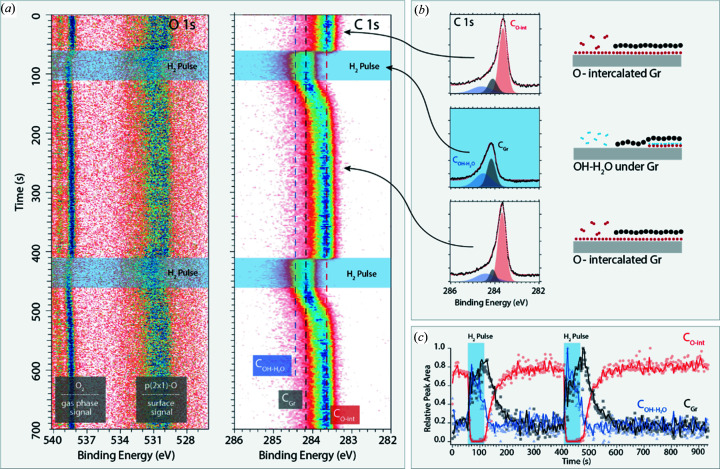
(*a*) Image plots of O 1*s* and C 1*s* spectra acquired with photon energies of 740 eV and 390 eV, respectively, and after background removal. (*b*) Examples of curve fits done after averaging 50–100 individual spectra and sketches explaining how graphene is intercalated (see the text for the details of the different components). (*c*) Time evolution of different components obtained after binning the data eight times (points). The solid lines are averages of 20 points.

In this example, we will demonstrate how gas pulses, the intense light at the HIPPIE beamline and fast acquisition of APXPS spectra can be used to follow a simple surface reaction – H_2_ oxidation – below graphene flakes on Ir(111). Fig. 9 ▸(*a*) shows image plots of the O 1*s* and C 1*s* core levels recorded *in situ* at 370 K and 1 mbar with 1 Hz and 4.2 Hz, respectively, on an Ir(111) surface half-covered by graphene flakes. While recording the XPS data the flakes were exposed to 10 s.c.c.m. O_2_ and two 50 s pulses of 9:1 H_2_:O_2_ s.c.c.m. marked in the figure.

Starting with the O 1*s* data, oxygen on the surface is signalled by a main component near 530 eV while the O_2_ in the gas phase is visible as a doublet component at 538.5 and 539.5 eV. The O-surface component is assigned to a *p*(2×1)-O phase both on the bare Ir(111) patches and under the graphene flakes in agreement with previous work (Grånäs *et al.*, 2012 ▸; Larciprete *et al.*, 2012 ▸). Even though a clear intensity reduction of the O_2_ gas-phase component is observed when the gas phase is diluted by the H_2_ pulse and a slight shift towards higher binding energies can be observed in the time-resolved data, it is difficult to say much about the H_2_ oxidation below the graphene flakes. One of the reasons is because the O 1*s* core level is weak and suffers from large background signal, and the other reason is because it is impossible to disentangle the oxidation on the bare Ir(111) patches and the oxidation below the graphene flakes. In contrast, the intense and sharp graphene component is very sensitive to the intercalated molecules or atoms below the flakes and a time evolution is clearly visible in the C 1*s* image plot. Between the H_2_ pulses – in the pure O_2_ flow – the curve fitting shown in panel (*b*) reveals that the C 1*s* spectrum is dominated by the component at 283.73 eV (C_O-int_ red component) matching well with graphene flakes intercalated by a *p*(2×1)-O phase (Grånäs *et al.*, 2012 ▸). In contrast, this component is fully absent in the centre of the H_2_ pulse. Instead, the curve fitting shows that the component is dominated by a component located at 284.15 eV (C_Gr_) and a broader component at 284.4 eV (C_OH-H2O_). The black component is assigned to non-intercalated graphene (Grånäs *et al.*, 2012 ▸), while the broad blue component is assigned to a mixed OH–H_2_O phase formed and partly trapped below the graphene flakes (Grånäs, 2014 ▸). Altogether, these results are clear evidence for H_2_ oxidation occurring below the graphene flakes, thereby titrating away the intercalated O atoms. As the mixed OH–H_2_O phase is denser than the *p*(2×1)-O phase (Grånäs, 2014 ▸) part of each graphene flake will re-laminate to the underlying Ir(111) surface as a result of the conversion of O atoms to OH and H_2_O molecules. In essence, this is an effect of the attractive hydrogen bonds formed in the mixed OH–H_2_O phase.

In Fig. 9 ▸(*c*) we plot the time evolution of the intensity of the different components. The semi-transparent data points were recorded with 4.2 Hz and binned eight times, while the solid lines are averaged over 20 data points. Interestingly, the oxidation proceeds very fast and within 7 s all O atoms below the graphene flakes are converted to OH or H_2_O molecules. In contrast, the re-intercalation by O_2_ is a slow process with a time constant, τ, of ∼25 s [obtained from fitting to 1 − *A* exp(*t*/τ) and denoting the time it takes to reach 63% of the saturation of the C_O-int_ component].

To conclude, this suggests a picture in which the small hydrogen molecules diffuse fast under the graphene flakes, react with O atoms and form OH and H_2_O molecules. In contrast, the subsequent removal of mixed OH–H_2_O phase and O intercalation proceed much slower with longer timescales. The example presented here demonstrates how the sharp and intense C 1*s* peak of graphene can be used as an additional signal to study heterogeneous chemistry with a time resolution of seconds and sub-seconds. Further, the example illustrates how transient gas supply together with a bright light source and an efficient electron analyser can be used to get insights into how quickly phase changes occur on surfaces and how this affects their catalytic properties – a theme we currently push at the HIPPIE beamline.

### 3.2. Electrochemistry   

The electrochemistry setup is demonstrated using a three-electrode Li-ion battery (LIB) setup where the material of the working electrode (WE) and the electrolyte are monitored during the first charge and discharge. The LIB comprised a sputter-deposited thin film of LiCoO_2_ (used as a WE), a composite Li_4_Ti_5_O_12_ counter-electrode and a Li-metal reference electrode (RE). The electrodes were immersed in an electrolyte with 1 *M* LiClO_4_ in propyl­ene carbonate and by using the dip-and-pull approach a thin meniscus was formed whereas both the LiCoO_2_ material and the electrolyte could be monitored simultaneously. A potentiostat (Biologic SP150) was then used to charge and discharge the LIB in the measurement position using fixed potential steps during which APXPS measurements were conducted (using a photon energy of 1800 eV). After each potential change the current was allowed to stabilize below 0.1 µA from the spike value of several µA before starting XPS measurements (∼1.5 h).

**Figure 10:**
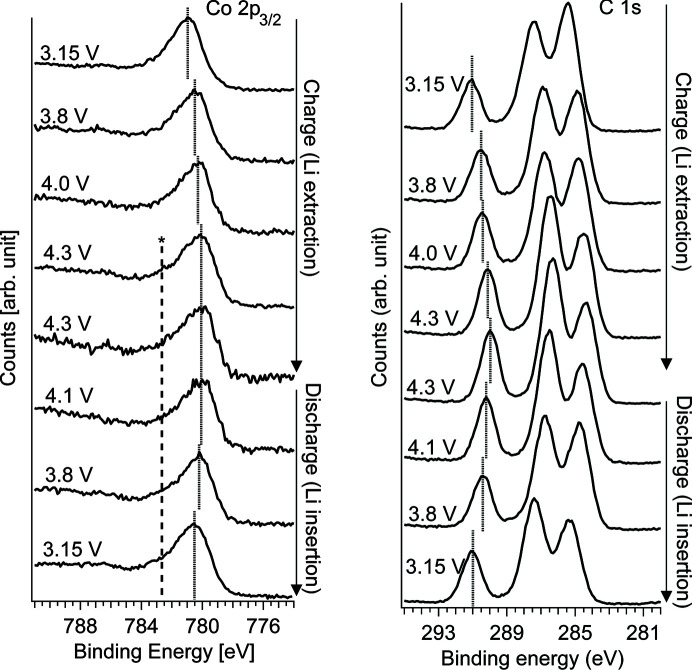
Left: Co 2*p*_3/2_ during the charge and discharge. The asterisk indicates where peak broadening is present. Right: C 1*s* spectra during the first charge and discharge. Dotted line indicates the peak of the carbonate group in the electrolyte solvent.

**Figure 11:**
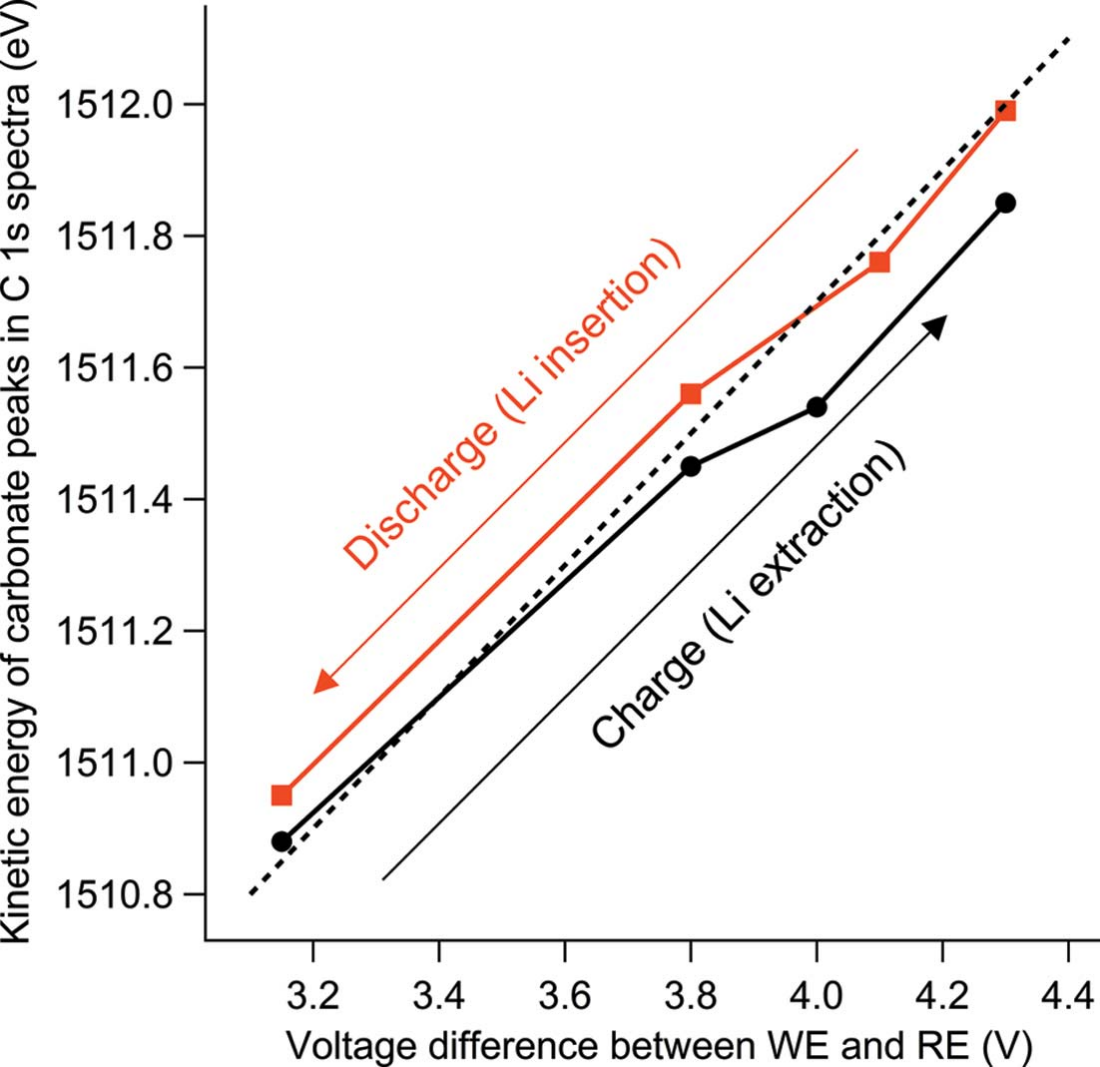
Peak position of the carbonate group in the electrolyte [deduced from Fig. 10 ▸ (right)] as a function of voltage difference between the working electrode (WE) and the reference electrode (RE). The dashed line represents the unity slope during the first charge and discharge of the battery.

In Fig. 10 ▸ (left) the Co 2*p*_3/2_ photoemission line is presented and the peak position is shifted to higher kinetic energies by about 1 eV during charge and shifted back to lower kinetic energies during discharge (by approximately 0.5 eV). Also, a small broadening [indicated by the dashed line (*) in Fig. 10 ▸ (left)] emerges during the first charge and then remains throughout the discharge. The C 1*s* spectra in Fig. 10 ▸ (right) represent the electrolyte at the interface with three peaks corresponding to three different carbon environments in the propylene carbonate molecule. The peak position of the carbonate peak [indicated by the dotted lines in Fig. 10 ▸ (right)] in each spectrum is presented in Fig. 11 ▸ as a function of WE potential versus Li^+^/Li (the RE). The carbonate peak position shifts approximately 0.8–0.9 eV per applied volt and there is an offset between charge and discharge of about 0.1 eV.

During charge (Li extraction) of LiCoO_2_ both cobalt and oxygen have been shown to partially oxidize (Kellerman *et al.*, 2006 ▸), and in the Co 2*p* photoemission spectra this results in a minor peak shift together with a broadening of the main peak and also an increase in the satellite (at about 8 eV higher kinetic energy than the main peak) (Dahéron *et al.*, 2008 ▸). The results presented herein confirm the broadening of the main peak whereas the satellites in the presented samples have a different appearance [possibly due to a slightly different stoichiometry (Dupin *et al.*, 2001 ▸)] and are less defined to allow for a detailed analysis. The shifting peak position of the Co 2*p*_3/2_ in the presented *operando* APXPS series reveals that the active material of the WE is undergoing a change due to the Li extraction and insertion. This shift could be related to the change in electronic structure since, during Li extraction, LiCoO_2_ changes from a semiconductor to a more metallic state (Kellerman *et al.*, 2006 ▸). During Li insertion (discharge) the peak position Co 2*p*_3/2_ shifts back although not to its original position and the peak broadening remains, indicating that the material does not completely return to the pristine state. The electrolyte peak position shifts less than the ideal 1 eV per V which suggests that a resistance is present in the cell, shifting the electrolyte potential less than the applied voltage difference. Also, the offset between charge and discharge indicates that the direction of the current possibly could affect interfacial mechanisms.

### 3.3. Adsorption of carbon monoxide on a Pt(111) surface at mbar pressures studied by PM-IRRAS and APXPS   

**Figure 12:**
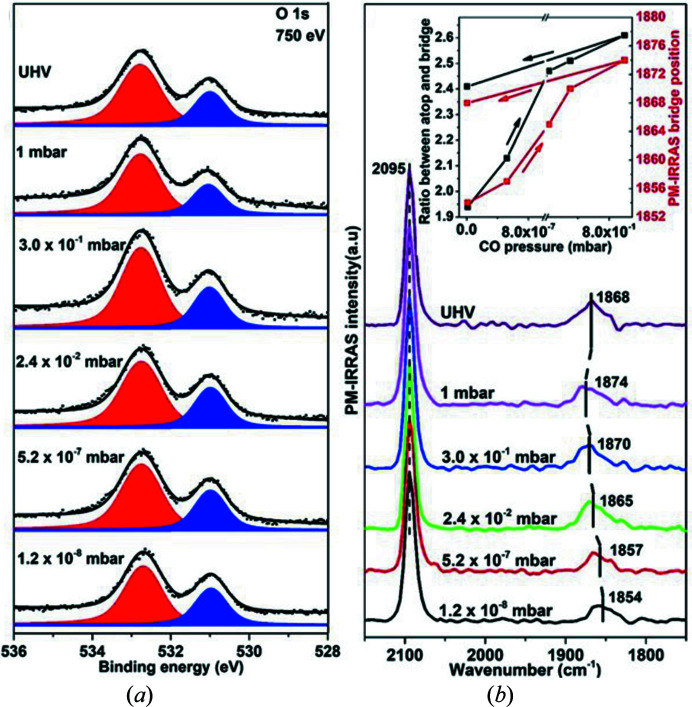
Simultaneous *in situ* (*a*) O 1*s* XPS and (*b*) PM-IRRAS spectra from Pt(111) as a function of CO pressure. The O 1*s* spectra were collected at *h*ν = 750 eV. The inset in (*b*) shows the correlation between the ratio of the O 1*s* on-top and bridge peak areas (left axis).

The *in situ* XPS and PM-IRRAS results were collected using the PM-IRRAS setup described above, with a spectral resolution of 8 cm^−1^. The Pt(111) crystal was cleaned by a standard sputtering–annealing procedure. Following CO introduction into the analysis chamber the O 1*s* XP spectra [Fig. 12 ▸(*a*)] show CO adsorbed in the expected on-top (532.7 eV) and bridge (531 eV) positions of Pt(111) (Knudsen *et al.*, 2016 ▸; Björneholm *et al.*, 1994 ▸). The on-top/bridge O 1*s* peak intensity ratio was calculated after fitting all the XP spectra. The evolution of the ratio as a function of CO pressures is shown as an inset in Fig. 12 ▸(*b*) (black curve). As expected, the on-top/bridge intensity ratio increases with CO pressure, which indicates that the surface accommodates an increasing coverage of on-top CO at higher CO pressures. Fig. 12 ▸(*b*) shows the simultaneously collected *in situ* PM-IRRAS spectra. The indicated peak positions reflect the centre of the integrated peak areas. Two absorption bands are observed at 2095 cm^−1^ and 1854 cm^−1^ at a CO partial pressure of 1.2 × 10^−8^ mbar, consistent with CO adsorption on Pt(111) on-top and bridge sites, respectively (Carrasco *et al.*, 2009 ▸, 2012 ▸). The position of the bridge component blue-shifts with increasing CO pressure. This blue shift has previously been interpreted as a signature for the formation of a compressed high-coverage *c*(4×2) CO layer on the surface and is interpreted as a result of an increased adsorbate–adsorbate repulsion (Carrasco *et al.*, 2009 ▸). The formation of the high-coverage CO phase changes the relative coverage of the CO adsorbed in on-top and bridge sites. In the inset of Fig. 12 ▸(*b*) this change in relative coverage is correlated with the IR position of the CO bridge band. At increasing bridge-to-on-top ratio, a blue shift in the CO bridge position is observed. Upon evacuation of the analysis cell, the CO on-top-to-bridge O 1*s* intensity ratio decreases slightly due to CO desorption from the surface. The decrease in CO bridge surface coverage is immediately observed in PM-IRRAS as a red shift in the CO bridge position.

## 4. Conclusion   

The HIPPIE beamline at the 3 GeV ring of Sweden’s national synchrotron radiation facility MAX IV Laboratory is a unique experimental system which allows users to employ the powerful APXPS technique for a wide range of scientific problems in various fields. Its high photon flux over a wide range of photon energies (250–2200 eV) at high resolving power (more than 10000 and up to a maximum 32000) allows quick XPS and NEXAFS (near-edge X-ray absorption fine structure) measurements at pressures of up to 30 mbar. A variety of exchangeable cells enables the use of different complex environments; this would not be possible in a single/fixed setup. A fully automated gas delivery and analysis system in combination with a fast detector allows studies of the kinetics of catalytic and other surface reactions with ms time resolution; the high flux at energies above 1500 eV and up to the P *K* edge and the availability of the EC cell allow studies of the solid–liquid interface under full electrochemical control; finally, a liquid microjet insert makes it possible to study surfaces of liquids and their interface with gas using a single instrument.

## Figures and Tables

**Table 1:** Beamline details

Beamline name	HIPPIE
Source type	EPU53
Mirrors	Au coated
Monochromator	cPGM
Energy range (keV)	0.25–2.2
Wavelength range (Å)	50–5.5
Beam size (µm)	*ca* 25 × 60
Flux (photons s^−1^)	>10^12^ (resolving power 12000–6000)
